# Artificial Intelligence in Pulmonary Hypertension: Advancements, Opportunities, and Challenges

**DOI:** 10.3390/jcm15218707

**Published:** 2026-11-07

**Authors:** Xinyi Li, Linyue Zhang, Li Zhang, Yuji Xie, Mingxing Xie, Mengmeng Ji, Qing He, Zhenni Wu, Shuxuan Qin, Yuman Li

**Affiliations:** 1 Department of Ultrasound Medicine, Union Hospital, Tongji Medical College, Huazhong University of Science and Technology, 1277 Jiefang Avenue, Wuhan 430022, China; 2 Clinical Research Center for Medical Imaging in Hubei Province, Wuhan 430022, China; 3 Hubei Province Key Laboratory of Molecular Imaging, Wuhan 430022, China; 4 Key Laboratory of Biological Targeted Therapy, Huazhong University of Science and Technology, Ministry of Education, Wuhan 430022, China

**Keywords:** pulmonary hypertension, artificial intelligence, machine learning, deep learning, diagnosis

## Abstract

Pulmonary hypertension (PH) is a progressive cardiopulmonary disorder associated with high morbidity and mortality. Delayed diagnosis, heterogeneous disease phenotypes, and variable treatment responses remain major clinical challenges. Artificial intelligence (AI), including machine learning and deep learning, has emerged as a promising tool for improving PH management. AI-based models have demonstrated promising performance in screening, diagnosis, classification, risk stratification, and prognosis prediction using multimodal data such as electrocardiography, chest radiography, computed tomography, cardiac magnetic resonance imaging, echocardiography, and electronic health records. Furthermore, AI may support individualized risk assessment, treatment-response prediction, early identification of right ventricular deterioration, and longitudinal remote monitoring. Emerging technologies, including explainable AI, federated learning, large language models, and digital twins, may further advance precision medicine in PH. Despite these advances, data quality, interpretability, privacy, and clinical implementation of AI remain challenges. This review summarizes current applications, opportunities, and challenges of AI in PH and discusses future directions for AI-assisted precision healthcare.

## 1. Introduction

Pulmonary hypertension (PH) is a progressive and heterogeneous cardiopulmonary disorder characterized by elevated pulmonary vascular resistance and increased right ventricular afterload, ultimately leading to right heart failure and premature death. Despite substantial advances in targeted therapies, delayed diagnosis remains a major challenge because of nonspecific early symptoms and the lack of sensitivity for detecting subclinical PH using conventional screening methods [1].

Artificial intelligence (AI), including machine learning (ML), deep learning (DL), natural language processing (NLP), and emerging foundation models, offers new approaches for integrating complex and multimodal medical data. By identifying nonlinear relationships and latent disease-related patterns across imaging, physiological signals, electronic health records, hemodynamic measurements, biomarkers, and molecular data, AI may support earlier disease recognition, more consistent classification, and improved risk assessment in PH [2].

Current applications span the clinical pathway. Deep-learning models applied to chest radiography and electrocardiography have shown promise for PH detection and early identification [3,4,5,6,7,8,9,10,11,12,13,14,15], while AI-assisted CT, cardiac magnetic resonance, and echocardiography enable automated quantification, phenotyping, and assessment of RV structure and function [16,17,18,19,20]. Beyond diagnosis, data-driven approaches are being explored for refined phenotyping, prognosis, treatment-response prediction, and detection of RV deterioration, with the aim of supporting more individualized clinical decision-making. However, many existing studies are retrospective, involve limited or selected cohorts, and lack robust external or prospective validation, raising concerns regarding generalizability, interpretability, bias, data privacy, and clinical integration.

**Figure 1:**
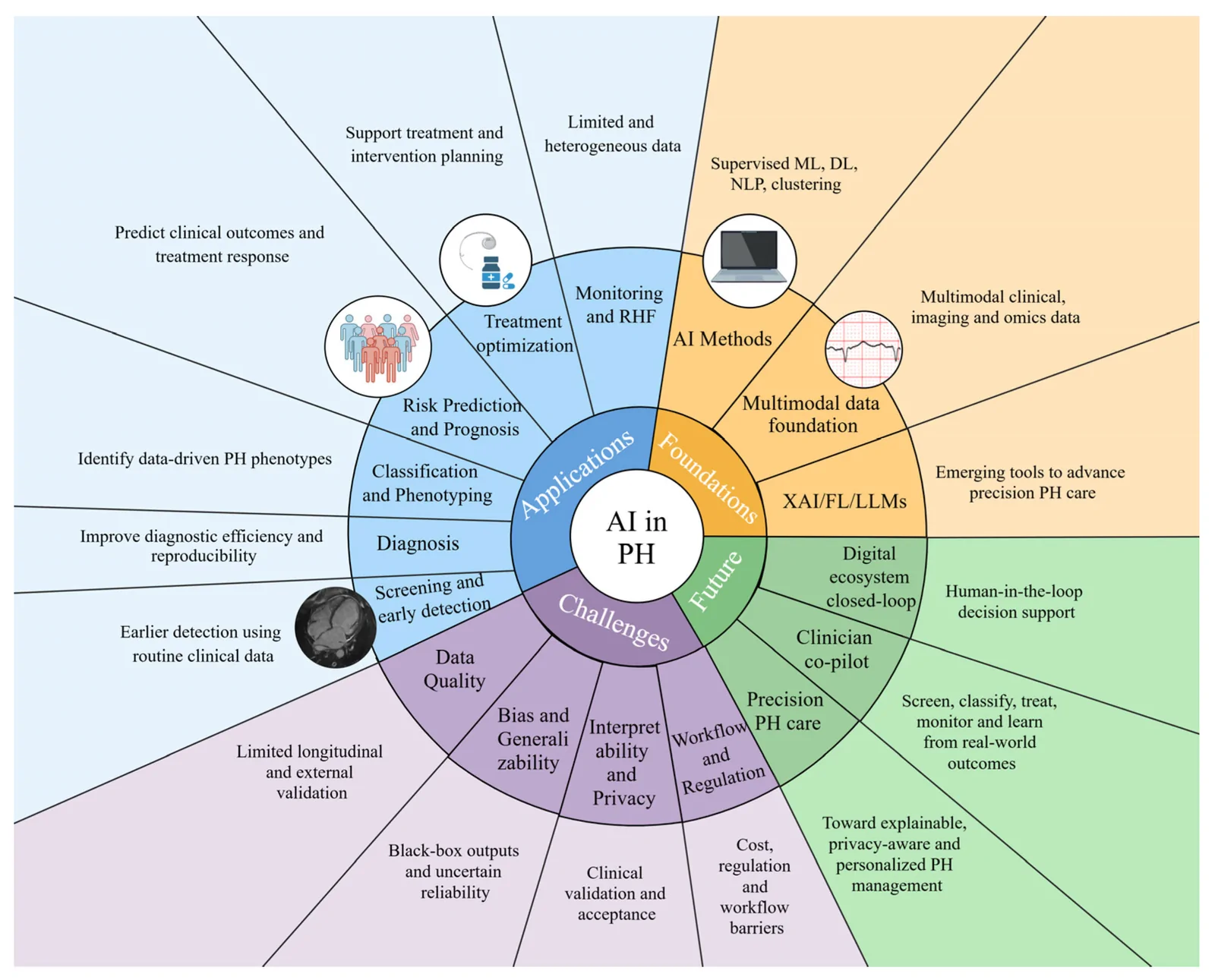
AI-assisted pulmonary hypertension care across the clinical continuum. Blue, yellow, purple, and green sectors represent applications, AI methods, challenges, and future directions, respectively.

This review places greater emphasis on the transition from individual AI applications toward precision and clinically integrated PH care. Specifically, we focus on three interrelated areas: (1) AI-driven precision phenotyping beyond conventional PH classification through the integration of clinical, imaging, hemodynamic, biomarker, and multi-omics data; (2) multimodal AI that integrates complementary data sources to support diagnosis, risk stratification, treatment-response prediction, and longitudinal monitoring; (3) clinical translation and implementation, including external validation, explainability, workflow integration, privacy-preserving learning, and collaboration between clinicians and AI systems. Accordingly, this review examines current and emerging applications of AI across the entire PH care pathway, from early detection and diagnosis to refined phenotyping, risk stratification, treatment optimization, and longitudinal disease management, and discusses how these advances may contribute to an integrated AI-assisted precision healthcare framework for PH (Figure 1).

## 2. Methods

A structured literature search was conducted to identify studies on AI applications in PH. PubMed and Google Scholar were searched for publications from January 2010 to January 2026, supplemented by manual screening of references from relevant studies and reviews.

Search terms combined PH-related keywords, including “pulmonary hypertension,” “pulmonary arterial hypertension,” “CTEPH,” “right ventricular dysfunction,” and “right heart failure,” with AI-related terms such as “artificial intelligence,” “machine learning,” “deep learning,” “neural network,” “natural language processing,” “clustering,” “phenomapping,” and “radiomics”. Additional terms covered clinical applications and data modalities, including screening, diagnosis, classification, phenotyping, prognosis, treatment response, electrocardiography, chest radiography, computed tomography, echocardiography, cardiac magnetic resonance, electronic health records, biomarkers, and omics. Boolean operators (“AND” and “OR”) were used to construct the search queries.

Titles and abstracts were screened first, followed by full-text review of potentially relevant articles. Studies were included if they evaluated AI-based methods in PH screening, diagnosis, classification, risk stratification, prognosis, treatment, right-heart assessment, or monitoring. Original studies, systematic reviews, meta-analyses, and relevant methodological reports were considered. Editorials, letters, case reports, studies without substantive AI methods, and studies primarily focused on other diseases were excluded.

Because this study was designed as a narrative review rather than a systematic review or meta-analysis, formal study-count tracking and risk-of-bias assessment were not performed. Study selection was guided by clinical relevance and methodological informativeness. Priority was given to original studies that directly evaluated AI methods in PH-related populations, clearly defined the clinical task and reference standard, reported quantitative performance or clinically relevant outcomes, and contributed distinct evidence regarding diagnosis, phenotyping, prognosis, treatment response, or implementation. When multiple studies addressed similar questions, greater emphasis was placed on larger cohorts, independent external validation, clinically robust reference standards, and more recent evidence. Studies with insufficient methodological detail, limited relevance to PH, or substantial overlap with previously reported cohorts were not emphasized. The selected literature was synthesized according to major clinical applications, methodological advances, limitations, and future directions of AI in PH.

## 3. Techniques and Methods of Artificial Intelligence

### 3.1. Supervised Machine Learning

Supervised learning uses labeled data, in which each training example contains input features and a predefined output label or target. In PH research, labels may include the presence or absence of PH, PH subtype, invasive hemodynamic measurements, treatment response, hospitalization, clinical worsening, or mortality. Supervised algorithms learn the mapping between predictors and outcomes and then apply this learned relationship to unseen patients. Common approaches include logistic regression, support vector machines (SVMs), random forests, gradient boosting, k-nearest neighbors, and Naive Bayes classifiers [21,22,23]. These methods are suitable for structured clinical data and tabular EHR variables, and can also be applied to radiomic or imaging-derived features.

### 3.2. Semi-Supervised, Weakly Supervised, Self-Supervised, and Transfer Learning

PH datasets usually contain a small number of well-labeled cases and a much larger pool of incompletely labeled or unlabeled observations. Semi-supervised learning addresses this problem by combining labeled and unlabeled data, allowing the model to use expert-confirmed cases while also learning the broader structure of the data set [23]. Weakly supervised learning is useful when labels are noisy, incomplete, or less precise than the desired output. For example, image-level labels or report-derived labels may be used when pixel-level segmentation or catheter-confirmed labels are unavailable. Self-supervised learning creates pretext labels directly from the data and learns transferable representations without manual annotation; this strategy underpins many modern foundation models [23]. Transfer learning and few-shot learning adapt models trained on large external datasets to smaller PH-specific datasets, which is important for rare diseases, pediatric PH, and underrepresented subgroups. These approaches can reduce annotation burden, but they may transfer bias from source populations or imaging protocols if domain shift is not assessed.

### 3.3. Unsupervised Learning, Dimensionality Reduction, and Phenomapping

Unsupervised learning identifies structure in unlabeled data without predefined outcomes. This approach is especially valuable in PH because guideline groups do not fully capture the heterogeneity of right ventricular adaptation, comorbidity burden, molecular mechanisms, or treatment response. Clustering algorithms such as k-means, hierarchical clustering, spectral clustering, affinity propagation, and mean shift group patients by similarity and can reveal clinically meaningful phenotypes or endotypes [23]. Dimensionality reduction methods, including principal component analysis, t-distributed stochastic neighbor embedding, locally linear embedding, and independent component analysis, simplify high-dimensional imaging, physiological, or omics datasets while preserving key information [23]. Unsupervised findings should be interpreted as hypothesis-generating unless replicated in independent cohorts and linked to biological plausibility or clinical outcomes.

### 3.4. Deep Learning and Neural Network Architectures

Deep learning uses neural networks with multiple layers to learn hierarchical features directly from raw or minimally processed data [24]. Recurrent neural networks and long short-term memory networks can model sequential data such as ECG waveforms, longitudinal EHR trajectories, wearable sensor signals, or serial follow-up measurements. Autoencoders learn compressed representations and can be used for anomaly detection, denoising, or unsupervised feature extraction, whereas generative adversarial networks can support data augmentation, image harmonization, and domain adaptation [23]. Transformer architectures use attention mechanisms to model long-range dependencies and have become central to modern NLP, large language models, and increasingly multimodal AI [25]. In model development, loss functions such as mean squared error, binary cross-entropy, and categorical cross-entropy are selected according to the task, while optimization commonly relies on stochastic gradient descent, Adam, and learning-rate scheduling. Performance should be reported with clinically appropriate metrics, including sensitivity, specificity, accuracy, precision, recall, F1-score, calibration, and area under the receiver operating characteristic curve.

### 3.5. Natural Language Processing, Generative AI, and Foundation Models

Natural language processing (NLP) enables AI systems to process unstructured text such as clinic notes, radiology reports, catheterization reports, discharge summaries, referral letters, trial eligibility criteria, and patient messages. Earlier NLP systems relied on rule-based or task-specific models, whereas contemporary generative AI and large language models are commonly based on large-scale pretraining and Transformer architectures. BERT, GPT, and related models demonstrate how pretraining can support question answering, summarization, information extraction, and few-shot learning [26,27]. Foundation models and generalist medical AI aim to provide reusable representations across tasks and modalities, potentially supporting multimodal PH workflows that combine text, imaging, waveforms, and structured clinical variables [28]. However, generated outputs can be inaccurate, incomplete, or overconfident. Therefore, LLM-based tools require source verification, clinician oversight, privacy protection, and prospective safety evaluation before clinical deployment.

## 4. Application of AI in the Diagnosis and Classification of Pulmonary Hypertension

### 4.1. Diagnosis of Pulmonary Hypertension

#### 4.1.1. Chest Radiography

PH is a severe cardiopulmonary condition characterized by elevated pulmonary arterial pressure. Delayed diagnosis of PH significantly affects patient prognosis. Chest X-ray (CXR) is a simple, inexpensive, and widely accessible imaging modality that is useful for PH screening, supporting diagnosis, and assessing disease severity. Although interpretation of conventional chest radiographs is subject to interobserver variability and early PH may have subtle or atypical radiographic findings, CXR can serve as a first-line screening tool and support referral for further examinations (e.g., echocardiography or right heart catheterization). The accessibility of CXR makes it particularly useful for preliminary PH screening in regions with limited medical resources [3]. With the rapid development of AI technology, CXR-based models for PH detection continue to emerge, further expanding the potential clinical applications of CXR in PH diagnosis.

In recent years, AI, particularly convolutional neural network (CNN)-based deep learning, has shown promise for noninvasive screening and assisted diagnosis of PH using CXR. Zou et al. [4] evaluated ResNet50, Xception, and Inception V3 in 762 participants from three institutions, with Inception V3 achieving AUCs of 0.970 and 0.967 in the internal and external test sets, respectively. The model also predicted pulmonary artery systolic pressure (PASP), with mean absolute errors of 7.45 and 9.95 mmHg, suggesting that deep learning can extract PH-related features beyond conventional visual assessment. However, the retrospective design and small external test set limited the robustness of the findings. AI-CXR has subsequently been evaluated across different PH subtypes and clinical settings. Kusunose et al. [5] evaluated exercise-induced PH in 142 patients with systemic sclerosis or related connective tissue disease. Adding the AI-predicted PH probability to clinical variables improved the AUC from 0.65 to 0.74, suggesting that deep learning may identify subtle pulmonary vascular abnormalities even on resting CXR. For pulmonary arterial hypertension (PAH), Imai et al. [6] developed a deep learning model that achieved an AUC of 0.988, with a sensitivity of 0.933 and specificity of 0.982, outperforming experienced clinicians. Similarly, Shimbo et al. [7] applied a CNN to 230 patients with systemic sclerosis undergoing CXR and right heart catheterization (RHC). The model achieved an AUC of 0.826 for PH detection, compared with 0.804 for cardiologists, and AI-detected PH was also associated with long-term outcomes, indicating potential value for both diagnosis and risk stratification. AI-CXR has also shown utility in congenital heart disease (CHD)-associated PAH. Han et al. [3] developed ResNet18-based models using 3255 preoperative CXR images, achieving AUCs of 0.948 for CHD and 0.778 for CHD-associated PAH. In a multi-reader, multi-case study, AI assistance increased radiologists’ mean AUCs by 0.096 and 0.066, respectively, highlighting its potential as an adjunct to clinical interpretation. Li et al. [8] further developed a ResNet model in 831 children with ventricular septal defects, achieving AUCs of 0.82 and 0.81 in the validation and independent test sets, respectively, compared with 0.65 for human observers. Class activation mapping showed that the model mainly focused on the pulmonary artery segment, providing some interpretability. Beyond single-modality approaches, multimodal AI may further improve PH detection. Kishikawa et al. [9] integrated ECG, CXR, and brain natriuretic peptide (BNP) data in an ensemble model. While the individual ECG, CXR, and BNP models achieved AUCs of 0.818, 0.823, and 0.724, respectively, their combination increased the AUC to 0.872, demonstrating the benefit of integrating complementary cardiopulmonary information. AI applications have also expanded to pulmonary venous hypertension (PVH). White et al. [10] developed a Transformer-based model in 1682 participants to grade PVH from CXR. AI-predicted PVH severity showed a stronger association with echocardiographic left ventricular diastolic dysfunction than conventional manual grading, suggesting improved standardization and reproducibility.

Overall, deep learning can extract clinically relevant pulmonary vascular and hemodynamic features from routine CXR and has demonstrated promising performance across PH screening, PAH detection, CHD-associated PAH, exercise-induced PH, and PVH assessment. However, the reported performance estimates should be interpreted cautiously because these studies addressed different clinical tasks and enrolled substantially different populations. Accordingly, these results should not be interpreted as directly comparable measures of model performance or as evidence that one model is superior to another. Most available studies are also retrospective and are limited by relatively small or selected cohorts, single-center designs, heterogeneous PH definitions or reference standards, and insufficient external validation. Larger prospective multicenter studies using standardized, preferably hemodynamic, reference criteria and independent external validation are required to establish the generalizability and clinical utility of AI-CXR for PH screening, decision support, and risk stratification.

#### 4.1.2. Electrocardiography

As a progressive pulmonary vascular disease, PH encompasses a heterogeneous group of disorders with multiple aetiologies. Diagnostic delays are associated with an increased risk of morbidity and mortality [29,30]; thus, this underscores the importance of early disease recognition [31,32] and the need to develop effective, noninvasive screening tools. Electrocardiogram (ECG), as one of the most widely used and standardized clinical tools in medicine, is inexpensive and performed regularly in primary care for patients [1]. However, ECG can only detect abnormalities in late-stage PH in most cases. In contrast, artificial intelligence, especially deep learning, is adept at identifying subtle patterns in 12-lead ECG that may indicate mild PH. Given the widespread use of ECG in primary care settings, ECG-based AI technology offers a key pathway for expanding the pool of patients requiring further evaluation [31].

By extracting latent information from voltage time-series data, AI-enabled ECG may improve PH detection. Several studies have demonstrated promising discriminatory performance of ECG-based AI models for PH diagnosis and risk assessment. DuBrock et al. [11] developed the PH-EDA algorithm, a CNN-based model using 12-lead ECG, which achieved an AUC of 0.92 for PH detection in the Mayo Clinic cohort and maintained high performance in external validation. Notably, the algorithm identified PH-related ECG signatures several years before clinical diagnosis, with particularly strong discrimination within 18 months before diagnosis, highlighting its potential for earlier disease recognition. Liu et al. [12] developed an AI-enabled ECG model that showed good performance for identifying patients with a high probability of PH using ten-fold cross-validation, with consistent results across subgroups stratified by age, sex, and major comorbidities. The model was also associated with mortality prediction, suggesting potential value beyond diagnosis alone. Similarly, Aras et al. [13] developed a deep learning model capable of detecting PH as well as clinically relevant subtypes, including pre-capillary PH and PAH. Importantly, the model retained favorable diagnostic performance even when limited ECG leads were used, indicating potential applicability to simplified or wearable ECG devices. Multimodal approaches may further enhance diagnostic accuracy. Liu et al. [14] integrated ECG and chest X-ray data into a deep learning framework, achieving AUCs of 0.8644 and 0.8734 in the internal and external validation sets, respectively. The negative predictive values (NPVs) exceeded 98% in both cohorts. However, because NPV is strongly influenced by disease prevalence, these values should be interpreted in the context of the populations in which the model was evaluated and should not be assumed to generalize to screening settings with different PH prevalence. Rather than independently excluding PH or avoiding RHC, such a model may be more appropriately considered as a screening or triage tool to identify patients who may warrant further clinical and echocardiographic evaluation. At the population level, Kalmady et al. [15] compared ResNet-based deep learning models with XGBoost models for ECG-based cardiovascular diagnosis and found that both approaches performed well, although deep learning generally showed superior predictive performance.

Overall, AI-enabled ECG can capture subtle electrical signatures associated with pulmonary vascular and right ventricular abnormalities that may not be apparent on conventional ECG interpretation. These models have shown potential for PH detection, subtype classification, prognostic assessment, and earlier disease recognition, while multimodal integration with imaging may provide complementary diagnostic information. However, clinical utility in screening cannot be determined by AUC alone and should also consider sensitivity, specificity, positive and negative predictive values, and the prevalence of PH in the intended population. In particular, predictive values derived from selected or enriched study cohorts may differ substantially when models are applied to lower-prevalence primary-care or population-based screening settings. Accordingly, AI-assisted ECG should currently be considered a tool for risk enrichment and referral rather than a standalone method for excluding or confirming PH. Prospective studies in intended-use populations are needed to determine clinically appropriate thresholds and whether AI-guided screening improves referral efficiency and subsequent diagnostic evaluation.

#### 4.1.3. CT

Chest computed tomography (CT), particularly CT pulmonary angiography (CTPA), provides a comprehensive assessment of pulmonary vasculature, cardiac structure, lung parenchyma, and mediastinal lesions. It serves as a core non-invasive imaging modality for the evaluation, etiological screening, and phenotypic classification of PH, and is recommended by the ESC/ERS guidelines for the assessment of all patients with suspected PH [1]. In recent years, AI technology has shown increasing potential in medical image analysis. Addressing key challenges in CT image assessment in PH, such as time-consuming manual measurements, significant inter-observer variability, difficulty in identifying early signs, and insufficient accuracy in subtype classification, AI technology has enabled comprehensive technological breakthroughs across the entire workflow. These range from precise quantification of imaging biomarkers and automated etiological classification to non-invasive prediction of hemodynamics and risk stratification, providing novel tools for the precise diagnosis and treatment of PH.

Deep learning has expanded the role of CT in PH by enabling automated segmentation, quantitative imaging analysis, and data-driven disease classification. Several studies have expanded the role of AI-CT from structural assessment to phenotyping and risk stratification of PH. Sharkey et al. [16] developed a fully automated deep-learning model for semantic segmentation of the four cardiac chambers and major vessels on CTPA. The model demonstrated high segmentation accuracy for most structures in both internal and external test cohorts. Automated segmentation enables reproducible extraction of volumetric cardiovascular imaging biomarkers and may support quantitative assessment of cardiac remodeling and hemodynamic burden. Beyond cardiac segmentation, AI can also facilitate objective assessment of pulmonary vascular morphology. Xu et al. [17] used AI-based quantitative analysis to evaluate CT morphological differences between CTEPH and chronic thromboembolic disease (CTED). They found that pulmonary artery tortuosity progressively increased from controls to CTED and CTEPH, and was moderately correlated with mean pulmonary artery pressure and pulmonary vascular resistance. Combining pulmonary artery tortuosity with small-to-medium pulmonary arterial volume parameters further improved discrimination between CTED and CTEPH, suggesting potential value in differentiating diseases with overlapping conventional CT features. AI has also facilitated automated assessment of lung parenchymal abnormalities associated with PH. Sharkey et al. [18] developed a lung segmentation model for CTPA images that achieved a DSC of 0.99 in the internal cohort. Combined with texture analysis and classification methods, the model enabled identification and quantification of five major lung parenchymal patterns, providing an automated approach for phenotyping PH-related lung disease. In the context of pulmonary embolism-associated PH, Silva et al. [19] developed a CNN-LSTM hybrid model for automated pulmonary embolism detection and risk assessment using CTPA. In external validation, the model achieved a precision of 86% for positive cases and 69% for negative cases, with a high negative predictive value that may help reduce unnecessary invasive investigations. AI may also assist in PH subtype classification, which remains challenging due to overlapping CT manifestations among different PH groups. Cereser et al. [20] developed a machine learning model based on expert-defined CT features for PH classification. The model achieved an overall accuracy of 0.72, with relatively favorable classification performance for Group II PH (associated with left heart disease) and Group IV PH (CTEPH). Furthermore, the authors developed an educational tool that provides probability scores for different PH subtypes based on standardized CT features, potentially supporting non-specialist radiologists in initial etiological screening and classification.

Overall, AI-assisted CT has shown potential for automated segmentation, quantitative biomarker extraction, disease phenotyping, and PH subtype classification. However, the available studies address heterogeneous analytical and clinical tasks, ranging from anatomical segmentation and pulmonary vascular quantification to pulmonary embolism detection and PH subtype classification. Their reported performance metrics therefore reflect different endpoints and should not be directly compared. In addition, several studies remain limited by retrospective designs, selected populations, and insufficient independent external validation. Further prospective multicenter studies using standardized imaging protocols and clinically relevant reference standards are required to establish the generalizability and clinical utility of AI-assisted CT in PH.

#### 4.1.4. Cardiac Magnetic Resonance Imaging

Cardiac magnetic resonance (CMR), as a radiation-free and non-invasive imaging technique, can comprehensively assess cardiac morphology, function, myocardial perfusion and unique tissue characterization, providing rich objective data for the evaluation of PH [33]. However, the broader clinical adoption of CMR has been limited by several factors such as the time required for image interpretation, the extensive training needed to acquire expertise, and a consequent shortage of physicians skilled in CMR [34].

In recent years, advances in AI have expanded the diagnostic potential of CMR imaging in PH. Swift et al. developed a tensor-based machine learning model that extracted diagnostic features directly from short-axis and four-chamber cine CMR images without manual segmentation [35]. The model achieved an AUC of 0.92 for PAH diagnosis, with analysis completed within 10 s, demonstrating the potential of AI for rapid and automated CMR-based diagnosis. Lungu et al. combined CMR-derived hemodynamic parameters with indices of cardiac morphology and function and developed a random forest model for PH detection [36]. The model achieved a diagnostic accuracy of 92%, suggesting that integration of computational hemodynamics with conventional CMR parameters may improve noninvasive identification of PH. However, the relatively small cohort warrants further validation in larger populations. AI has also improved automated quantitative assessment of cardiac remodeling in PH. Alabed et al. developed a fully automated deep-learning tool for ventricular CMR measurements in patients with suspected PH [37]. Automated measurements showed good agreement with manual analysis, high scan-rescan repeatability, associations with invasive hemodynamics, and prognostic value for mortality. Alandejani et al. further developed an AI model for automated right atrial measurements using CMR [38]. The automated measurements showed higher inter-study repeatability than manual analysis, and were associated with invasive hemodynamics and mortality, supporting their potential role in standardized PH risk assessment. Collectively, these studies demonstrate the potential of AI-assisted CMR for rapid disease classification and reproducible quantitative assessment of cardiac remodeling in PH. Nevertheless, the available studies differ substantially in clinical tasks, study populations, outcome definitions, and validation strategies, and some were conducted in relatively small or selected cohorts. Reported diagnostic and quantitative performance should therefore be interpreted within the context of each individual study rather than compared directly across models. Further prospective multicenter validation is required before routine clinical implementation.

#### 4.1.5. Echocardiography

Echocardiography is the first-line non-invasive imaging modality for screening and evaluating PH. It provides real-time information on pulmonary artery pressure estimation, right atrial and right ventricular morphology, right ventricular systolic function, tricuspid regurgitation, interventricular septal configuration, and indirect signs of increased pulmonary vascular resistance. However, conventional echocardiographic assessment remains limited by operator dependence, variable image quality, inter-observer variability, and the incomplete reliability of pressure estimation based on tricuspid regurgitation velocity alone. These limitations are particularly important in primary care, pediatric cardiology, and resource-limited settings where experienced echocardiographers may not be readily available.

AI has shown considerable potential to improve the automation, reproducibility, and diagnostic performance of echocardiography in PH. Leha et al. developed machine learning models using routinely acquired echocardiographic parameters, with the best random forest model achieving an AUC of 0.87 for PH prediction [39]. By integrating multiple echocardiographic features rather than relying on a single parameter, this approach supports multiparametric assessment of PH probability. Liao et al. further developed a machine learning model based on parasternal short-axis echocardiographic images, which showed promising discriminatory performance in both derivation and external validation cohorts [40]. Its performance was also compared with conventional echocardiographic assessment within the study, suggesting that AI may capture imaging features complementary to routinely assessed parameters. These findings suggest that AI may identify subtle imaging features beyond routine visual interpretation. AI has also been applied to automated measurement and direct disease recognition. Salehi et al. evaluated an AI-based tool for automated tricuspid regurgitation jet velocity measurement in 1031 patients with suspected PH [41]. The system successfully measured tricuspid regurgitation jet velocity in 87% of echocardiograms and achieved diagnostic performance comparable to manual assessment, with an AUC of 0.88. Sun et al. developed an interpretable Chamber Attention Network for PAH diagnosis, which identified the right ventricle and right atrium as key diagnostic regions [42]. In infants with PAH after ventricular septal defect correction, Luo et al. developed a multi-modal AI model integrating echocardiography and chest radiography [43]. The multi-modal model showed higher performance than the corresponding single-modality models, with AUC, accuracy, and F1-score all exceeding 0.90 in internal and external testing. Beyond diagnosis, AI-assisted echocardiography has potential applications in risk stratification and treatment decision support. Jiang et al. [44] developed an interpretable machine learning model integrating echocardiographic, laboratory, and demographic variables for PH risk prediction, showing strong performance in internal and external validation. In chronic thromboembolic PH, Xi et al. [45] combined clinical and echocardiographic features to predict response to balloon pulmonary angioplasty, with the best model achieving an AUC of 0.865. Collectively, these studies indicate that AI-assisted echocardiography has potential applications in PH detection, automated quantification, risk stratification, and individualized treatment planning. However, these studies evaluated substantially different tasks, including image-based PH classification, automated TRJV measurement, multi-modal PAH detection, risk prediction, and treatment-response assessment, using different reference standards and validation strategies. Their reported performance metrics should therefore be interpreted within the context of the respective clinical tasks rather than directly compared across studies. Prospective multicenter studies using standardized reference criteria and independent external validation are still required to establish generalizability and clinical utility.

Taken together, these studies demonstrate the potential of AI across multiple diagnostic modalities in PH. However, their reported performance should be interpreted in the context of differences in study populations, clinical tasks, validation strategies, and, importantly, the reference standards used to define PH. Some models were evaluated against RHC-confirmed PH, whereas others relied on echocardiographic estimates, clinical diagnoses, diagnostic codes, or other study-specific labels; these endpoints are not interchangeable. (Table 1 summarizes the key methodological characteristics, reference standards, validation strategies, performance, and major limitations of representative studies).

### 4.2. General Classification of Pulmonary Hypertension Based on Existing Clinical Guidelines

According to the 2022 ESC/ERS guidelines, PH is hemodynamically defined by mPAP > 20 mmHg at rest, as measured by RHC. Pre-capillary PH is defined by mPAP > 20 mmHg, pulmonary arterial wedge pressure (PAWP) ≤ 15 mmHg, and pulmonary vascular resistance (PVR) > 2 Wood units (WU), whereas post-capillary PH is characterized by mPAP > 20 mmHg and PAWP > 15 mmHg. Post-capillary PH is further classified as isolated post-capillary PH when PVR is ≤2 WU and combined post- and pre-capillary PH when PVR is >2 WU [1]. Although echocardiography plays a central role in estimating the probability of PH, definitive hemodynamic diagnosis and classification require RHC. Within this hemodynamic framework, PH is not a single disease but a heterogeneous clinical syndrome arising from a wide range of cardiovascular, pulmonary, thromboembolic, congenital, and systemic conditions. Accordingly, PH is classified into five major clinical groups: pulmonary arterial hypertension, PH associated with left heart disease, PH associated with lung diseases and/or hypoxia, PH associated with pulmonary artery obstruction, and PH with unclear or multifactorial mechanisms [1]. This classification provides the foundation for diagnostic evaluation, treatment selection, and prognostic assessment. In clinical practice, however, accurate classification can be challenging because symptoms, imaging manifestations, and some hemodynamic characteristics may overlap across different PH groups.

AI may help address this challenge by integrating multidimensional information from routine clinical examinations, including imaging, echocardiography, clinical variables, and hemodynamic data. Its role is not to replace RHC or expert judgment, but to provide decision support for earlier stratification, more appropriate referral, and more consistent phenotyping. By extracting quantitative features that are difficult to assess reliably by visual interpretation alone, AI can complement guideline-based classification and improve the use of routinely available diagnostic data. Among imaging modalities, CT-based AI has shown particular value in PH phenotyping because CT can simultaneously assess pulmonary vessels, lung parenchyma, cardiac morphology, and chronic thromboembolic changes [18]. Quantitative CT analysis has been used to distinguish CTEPH from chronic thromboembolic disease by detecting subtle differences in the pulmonary vascular tree [17]. In addition, machine-learning models based on expert-defined contrast-enhanced CT features may assist non-specialist radiologists in classifying PH according to guideline-based categories [20].

Echocardiographic AI also contributes to PH classification. Models using routinely acquired echocardiographic and clinical variables have been developed to distinguish non-PH, pre-capillary PH, and post-capillary PH [46]. This is especially relevant because differentiating PAH from PH associated with left heart disease (PH-LHD) remains clinically challenging. Machine-learning approaches designed specifically to separate PH-LHD from PAH further support the potential of AI-assisted echocardiography in guideline-based classification [47]. Nevertheless, AI-derived classifications based on non-invasive imaging or other surrogate features should not be considered equivalent to invasive hemodynamic classification. Taken together, these findings suggest that AI may serve as an upstream decision-support tool to improve early classification and referral, while definitive diagnosis and treatment decisions should continue to rely on expert assessment and RHC.

### 4.3. Refined, Precise Classification of Pulmonary Hypertension

Beyond conventional guideline-based classification, data-driven approaches are beginning to refine PH phenotyping by revealing heterogeneity that is not fully captured by the five clinical groups. This is particularly relevant because patients assigned to the same guideline category may still differ markedly in clinical course, treatment response, comorbidity burden, right ventricular adaptation, and long-term prognosis. Integrating clinical records, imaging, hemodynamics, biomarkers, and multi-omics data may therefore help move PH classification from broad diagnostic labels toward more biologically and prognostically meaningful subgroups [48,49]. Biomarker-based and omics-based studies also support this view, as genomic, transcriptomic, proteomic, metabolomic, microRNA, exosome, and cell-free DNA data are increasingly being explored to clarify disease mechanisms and improve prognostic assessment in PAH [50].

AI has increasingly been applied to identify clinically meaningful phenotypes in PH. Parikh et al. [51] used cluster analysis in the FREEDOM trials to identify four PAH phenotypes with distinct baseline characteristics and differential responses to oral treprostinil. These findings suggest potential treatment-response heterogeneity across phenotypes but remain exploratory and require independent validation before being used for treatment selection. Sonnweber et al. [52] combined supervised Elastic Net modeling with medoid clustering to derive a seven-variable mortality signature and distinct PAH risk phenotypes. The high-risk cluster was characterized by older age, impaired cardiac output, higher pulmonary vascular resistance, worse exercise capacity, and poorer prognosis. Similar clustering approaches have identified high-risk PH phenogroups characterized by combined kidney, heart, and interstitial lung disease comorbidity [53] and have been explored for long-term mortality prediction [54].

Molecular and immune profiling further refines PH phenotyping. Unsupervised transcriptomic analysis identified three idiopathic PAH endophenotypes with distinct molecular signatures and prognoses [55]. Sweatt et al. [56] identified four immune phenotypes based on circulating cytokines and chemokines, which differed in clinical risk and 5-year transplant-free survival. This concept has also been explored in the context of treatment selection. For example, the SATISFY-JP study investigated IL-6-related immune phenotyping in relation to satralizumab therapy [57]. Such biomarker-defined treatment-response phenotypes remain investigational and require prospective validation before they can be used to guide routine therapeutic decisions. Metabolomic analysis from PVDOMICS further demonstrated distinct lipid and fatty acid metabolic profiles between connective tissue disease-associated PAH and idiopathic PAH, suggesting biological mechanisms underlying differences in prognosis and treatment response [58]. Emerging multi-omics approaches have further implicated microbiome-associated metabolites in PH phenotyping and disease mechanisms [59]. Recent bioinformatics and multi-omics studies have further expanded molecular phenotyping in PAH. Machine-learning analysis has been used to identify immune-related gene signatures with potential diagnostic relevance [60], while integrated multi-omics analyses have implicated mitochondrial electron transport chain dysfunction in PAH pathogenesis [61]. These findings illustrate how AI-assisted molecular profiling may help identify biologically distinct disease mechanisms, although their clinical utility requires further validation.

Imaging and hemodynamic approaches provide additional dimensions of precision phenotyping. MRI-based deep phenotyping has been used to distinguish adaptive from maladaptive right ventricular remodeling [62], while imaging-based analyses of metabolic pathways have provided further insights into right ventricular adaptation to PH [63]. Advanced hemodynamic clustering has also identified five right ventricular functional subphenotypes that crossed conventional World Symposium PH classifications [64]. Right ventricular functional assessment may also provide prognostic information in CTEPH, with three-dimensional echocardiographic measures of RV function associated with adverse clinical outcomes [65]. CT-based quantitative imaging and machine-learning approaches have also been used to characterize vascular morphology, predict hemodynamic endpoints, quantify right-to-left ventricular relationships, and support phenotyping in CTEPH [66,67,68,69,70,71]. Automated dual-energy CTPA perfusion analysis differentiated chronic thromboembolic pulmonary disease from chronic thromboembolic PH, and perfusion abnormalities were correlated with pulmonary vascular resistance and mean pulmonary artery pressure [72]. In COPD, machine-learned CT emphysema subtypes were associated with distinct MRI-derived hemodynamic profiles, suggesting that lung parenchymal phenotypes may contribute to cardiopulmonary heterogeneity in group 3 PH [73]. EHR-based phenotyping may further facilitate large-scale automated cohort identification and characterization [74].

Collectively, AI-driven phenotyping complements rather than replaces guideline-based PH classification. Current studies demonstrate that data-driven approaches can identify clinical, molecular, immune, imaging, and hemodynamic subgroups associated with differences in disease characteristics and prognosis. However, many reported phenotypes remain exploratory, and independent replication across external cohorts is limited. Importantly, associations between a phenotype and differential treatment response do not by themselves establish clinical utility for treatment selection. Prospective studies demonstrating reproducibility, treatment-by-phenotype interactions, and improved clinical outcomes are required before AI-derived phenotypes can guide individualized therapy. Table 2 summarizes the major phenotyping approaches explored in PH.

## 5. Application of AI in the Prediction, Prognosis, and Prevention of Pulmonary Hypertension

### 5.1. Early Prediction and Identification of Pulmonary Hypertension

AI may facilitate earlier identification of individuals with undiagnosed PH or at increased risk of developing PH. This is clinically relevant because early symptoms are often nonspecific and diagnostic delay remains common. By analyzing routinely available data, including ECG, EHRs, claims data, chest radiography, and echocardiography, AI may detect subtle signals of pulmonary vascular disease or right-heart remodeling before conventional clinical recognition.

ECG-based AI has shown particular promise for early detection. Kwon et al. [85] developed an interpretable model using 12-lead ECG and demographic data, achieving AUCs of 0.859 and 0.902 in internal and external validation, respectively. DuBrock et al. [11] further demonstrated that ECG-based AI could retain discriminatory ability months to years before clinical diagnosis, supporting its potential for identifying patients who may require further evaluation. Real-world clinical data provide another route for population-level case finding. Kogan et al. [86] developed an XGBoost model using EHR data that achieved an AUROC of 0.92 and remained informative more than 18 months before diagnosis. Claims-based machine-learning models have likewise been used to identify PAH before formal diagnosis [87,88,89]. Imaging-based approaches, including chest radiography and echocardiography, may further support early PH detection and referral [4,8,39,44,90,91].

Overall, AI may help shift PH care from symptom-driven recognition toward earlier risk identification by leveraging routinely collected clinical data. These tools may support surveillance and specialist referral, but should complement rather than replace confirmatory hemodynamic assessment.

### 5.2. Prognosis Determination

AI-based prognostic models can integrate clinical variables, imaging markers, hemodynamic parameters, biomarkers, and omics data to estimate clinically relevant outcomes such as mortality, transplantation, hospitalization, and clinical worsening. One example is the doctor-AI cooperative prognostic platform developed by Kheyfets et al. [81], which used random-forest modeling to estimate the 4-year risk of death or transplantation in PAH. By incorporating Shapley-based feature attribution, the model also provided patient-specific explanations of the variables contributing to predicted risk, illustrating how AI may combine prognostic discrimination with clinically interpretable risk assessment. This type of approach may be particularly useful in PH, where prognosis depends on interactions among functional capacity, right-heart function, hemodynamics, biomarkers, and comorbidities rather than on a single risk marker.

Imaging-derived AI markers provide an additional source of prognostic information. Dwivedi et al. [77] showed that AI-based quantification of fibrosis and radiologic disease severity on baseline CT could contribute to prognostic assessment in PH, extending CT analysis beyond conventional visual interpretation. CMR-based approaches are particularly relevant because right ventricular adaptation is a major determinant of outcome in PH. In newly diagnosed PAH, machine-learning features derived from CMR improved short-term mortality prediction and provided prognostic information beyond conventional clinical risk assessment [92]. Automated deep-learning CMR measurements have also shown good agreement with manual analysis and associations with invasive hemodynamic parameters and mortality [37]. Similarly, AI-derived right atrial measurements demonstrated high reproducibility and were associated with both catheterization-derived hemodynamics and mortality, supporting their potential use for standardized longitudinal risk assessment [38]. ECG-based AI may provide a more accessible complementary approach. Liu et al. [12] showed that an AI-enabled ECG model was associated not only with PH detection but also with mortality prediction, suggesting that latent electrical signatures may contain prognostic information beyond conventional ECG interpretation.

AI-derived measurements of right ventricular size, function, and remodeling should be interpreted within the pathophysiological framework of right ventricular-pulmonary arterial (RV-PA) coupling rather than as isolated imaging biomarkers. In PH, clinical deterioration reflects both pulmonary vascular load and the ability of the RV to adapt to that load. AI-based approaches have also been explored for noninvasive estimation of pulmonary artery pressure, supporting the potential role of data-driven models in hemodynamic assessment [93]. Computational approaches for acquiring right ventricular pressure-volume hemodynamics may further support quantitative assessment of RV function and RV-PA coupling in PH [94]. Accordingly, AI-assisted right-heart assessment may be most clinically meaningful when imaging-derived measures of RV remodeling are integrated with hemodynamic load, functional capacity, biomarkers, and clinically meaningful outcomes such as hospitalization, clinical worsening, transplantation, or mortality [37,38,62,64,65,76].

Machine learning may also refine risk stratification by identifying prognostically distinct patient profiles that are not fully captured by conventional categorical risk scores. Supervised feature selection combined with unsupervised clustering has been used to derive mortality-related signatures and clinically distinct PAH phenotypes. In one approach, an Elastic Net model identified a seven-variable mortality signature incorporating age, six-minute walking distance, red blood cell distribution width, cardiac index, pulmonary vascular resistance, NT-proBNP, and right atrial area; subsequent clustering separated patients into risk phenotypes with different clinical characteristics and outcomes [52]. High-risk phenotypes were characterized by older age, impaired cardiac output, higher pulmonary vascular resistance, reduced exercise capacity, and poorer prognosis. Clustering-based approaches have also been explored for longer-term mortality prediction [54], while Kanwar et al. [95] applied Bayesian analysis to PAH risk stratification, demonstrating an alternative probabilistic framework for integrating multiple clinical predictors. The latter may be particularly useful because Bayesian models can express individualized risk estimates while accommodating complex relationships among prognostic variables.

Collectively, these studies suggest that AI may complement established PH risk-assessment frameworks by integrating multimodal information and identifying complex prognostic patterns. However, improved discrimination alone does not establish clinical usefulness. For prognostic models intended to support treatment escalation or follow-up decisions, calibration is essential because it reflects the agreement between predicted and observed risk, whereas a model with a high AUC may still systematically overestimate or underestimate absolute risk. External validation across independent centers and clinically relevant PH subgroups is also necessary to assess generalizability. Importantly, AI-based models should be compared directly with established clinical risk-assessment approaches and should demonstrate incremental value beyond routinely available risk scores rather than performance in isolation. Ultimately, prospective impact studies are needed to determine whether AI-assisted risk estimates change clinical decisions and improve patient outcomes.

The incremental value of AI should also be judged against established PH risk-stratification pathways rather than against no comparator. ESC/ERS risk assessment and REVEAL-based models already combine validated clinical, functional, biomarker, and hemodynamic variables to support treatment and follow-up decisions. Therefore, new AI models should demonstrate added value beyond these frameworks, for example through improved calibration, clinically meaningful reclassification, longitudinal prediction, or integration of high-dimensional imaging and multimodal data not adequately represented in conventional scores. At present, direct head-to-head comparisons with ESC/ERS or REVEAL-based approaches remain limited, and evidence that AI-guided risk assessment improves treatment decisions or outcomes beyond established tools remains insufficient [1,81,92].

### 5.3. Prevention

In PH, prevention encompasses early detection, identification of patients at risk of deterioration, and reduction in adverse outcomes. For primary prevention and early detection, AI may help identify individuals who require further PH evaluation before a definitive diagnosis is made. This is particularly relevant because PAH is rare, early symptoms are nonspecific, and current screening is mainly focused on selected high-risk groups such as systemic sclerosis, portal hypertension, BMPR2 mutation carriers, and relatives of patients with heritable PAH [96]. ECG-based models have shown that routine 12-lead ECGs may contain PH-related patterns even months to years before diagnosis, supporting their potential use as a low-burden screening tool [11,85]. EHR-based and claims-based models further suggest that demographic information, diagnoses, procedures, prescriptions, laboratory tests, and healthcare-utilization patterns can help identify patients with possible undiagnosed PH before formal recognition [86,88,97].

AI may also support prevention and earlier intervention in high-risk populations. In systemic sclerosis, a proteomic random-forest model identified an eight-protein biomarker panel for distinguishing patients with and without PAH [78]. In chronic kidney disease, a machine-learning nomogram was developed to estimate the risk of developing PH during follow-up [98]. Machine-learning models have also been applied to very preterm infants with bronchopulmonary dysplasia to identify those at increased risk of BPD-associated PH, where early recognition may be particularly important [99]. Imaging and low-cost physiologic signals provide additional screening opportunities. Chest radiograph-based AI models have been used for PAH detection, congenital heart disease-associated PAH, and prediction of elevated pulmonary artery pressure in children with ventricular septal defects [3,4,5]. Digital-stethoscope phonocardiography has also shown potential as an accessible PH screening approach, particularly in settings where echocardiography is less readily available [100]. For secondary and tertiary prevention, AI has increasingly been applied to identify patients at risk of clinical deterioration or readmission. In pediatric PH, an explainable CatBoost model predicted 30-day unplanned readmission and identified clinically relevant factors including age, length of stay, congenital heart surgery, and discharge status [101]. Collectively, these approaches may help prioritize surveillance, guide follow-up intensity, and support earlier referral of high-risk patients to specialized PH centers.

Overall, AI-assisted prevention in PH is promising but remains at an early stage. Most available studies are retrospective and require prospective validation before routine clinical use. At present, AI should be regarded as a supportive tool for screening, triage, and risk stratification rather than a replacement for clinical judgment. Its future value will depend on whether these models can be integrated into real-world workflows and lead to measurable reductions in diagnostic delay, hospitalization, and disease-related mortality. The major AI applications across the PH clinical pathway are summarized in Table 3.

## 6. Opportunities and Challenges of AI Applications in the Clinical Practice of Pulmonary Hypertension Care

### 6.1. Opportunities for AI Application in the Clinical Practice of Pulmonary Hypertension Care

#### 6.1.1. Precision

One of the most immediate opportunities for AI in PH care is to improve the precision of clinical assessment. PH is a heterogeneous syndrome, and conventional evaluation often depends on fragmented information from imaging, hemodynamics, biomarkers, and clinical judgment. AI can help integrate these data and extract quantitative features that are difficult to capture through visual interpretation alone. In CT-based studies, AI has been used for pulmonary vascular segmentation, lung parenchymal assessment, and PH phenotype recognition, supporting more objective classification and treatment planning [18]. Echocardiographic AI can also refine PH assessment by combining multiple parameters rather than relying on a single pressure estimate. Machine-learning models have been used to distinguish non-PH, pre-capillary PH, and post-capillary PH, while automated tricuspid regurgitation jet velocity measurement showed diagnostic performance comparable to manual assessment [41,46].

Precision is not limited to measurement automation. Interpretable echocardiographic models, such as the Chamber Attention Network, can highlight right-heart regions that are clinically relevant to PAH diagnosis, making AI output more consistent with cardiovascular pathophysiology [42]. In addition, supervised and unsupervised learning methods have identified PAH risk signatures and patient clusters with distinct prognoses, suggesting that AI can capture heterogeneity within the same diagnostic category [52]. MRI-based deep phenotyping of the right ventricle further illustrates this value by distinguishing adaptive and maladaptive remodeling states, while clinical-data clustering has identified mortality-related PH phenotypes [54,62]. More broadly, standardized quantitative assessment of RV structure and function is important for characterizing right-heart adaptation and disease severity in PH [76]. These findings indicate that AI may help move PH care from broad guideline categories toward more quantitative, phenotype-driven, and individualized assessment.

#### 6.1.2. Penetration

Another important opportunity is the broader integration of PH screening into routine and primary care settings. Delayed diagnosis remains common because early symptoms are nonspecific and specialized tests such as echocardiography, CMR, and right heart catheterization are not always immediately available. AI can leverage low-cost and widely performed examinations, including chest radiography, ECG, digital auscultation, BNP testing, and electronic health records, to identify patients who should receive further evaluation.

CXR-based deep learning models have shown promising performance in detecting PAH or PH-related abnormalities from routine radiographs, with some models outperforming experienced clinicians and maintaining good performance in external testing [4,6,90]. ECG-based AI offers another scalable route because standard 12-lead ECGs are inexpensive and frequently obtained in outpatient and inpatient care. Several ECG-AI models have detected PH, elevated pulmonary pressure, and clinically important PH subtypes, and some retained predictive ability months to years before formal diagnosis [11,12,13,85]. Multimodal models combining ECG, chest X-ray, and BNP further improved diagnostic performance and helped physicians identify PH more accurately [9,14]. Digital stethoscope and heart-sound algorithms may also be useful in community or resource-limited settings, where they provide a portable and noninvasive screening option [100,102]. Therefore, AI may extend PH screening beyond specialist centers and help high-risk patients enter the diagnostic pathway earlier.

#### 6.1.3. Personalization

The long-term value of AI in PH lies in personalization. Current treatment decisions are still largely based on clinical group, risk status, and stepwise treatment response, but patients within the same PH group may differ substantially in biology, right ventricular adaptation, comorbidities, and therapeutic benefit. AI can help connect individual patient features with prognosis, phenotype, and treatment response. A doctor-AI cooperative platform using random forest modeling and Shapley value interpretation predicted 4-year death or transplantation risk in PAH and, more importantly, identified patient-specific variables driving the predicted risk [81]. This type of interpretable model may help clinicians understand why a patient is classified as high risk and which factors may deserve closer attention. Computational simulator models combined with invasive hemodynamic monitoring provide another path toward personalized care by allowing dynamic assessment of disease status and potential treatment effects [82].

Personalization is also supported by phenotype-based and biomarker-based studies. Elastic Net risk signatures and clustering methods have identified PAH phenotypes with different clinical profiles and outcomes [52]. Multi-omics and machine-learning studies have described hypoxia-related molecular subtypes, metabolomic differences between connective tissue disease-associated PAH and idiopathic PAH, and immune phenotypes with different transplant-free survival [56,58,79]. These approaches may eventually help match patients to targeted therapies or clinical trials. In treatment-response prediction, machine learning has been used to predict wogonin response in experimental PH and to estimate the efficacy of balloon pulmonary angioplasty in inoperable CTEPH using clinical and echocardiographic variables [45,83]. Beyond treatment-response prediction, computational approaches may also support therapeutic discovery and drug repurposing in PH. Network-based methods have been explored for systematic drug repositioning in PAH [103], while AI-integrated biosimulation platforms illustrate the potential to accelerate the repurposing and development of PH-related therapies [104]. Taken together, AI may provide a foundation for moving PH management away from a trial-and-error model toward more patient-specific, mechanism-informed care.

### 6.2. Challenges of AI Application in the Clinical Practice of Pulmonary Hypertension Care

#### 6.2.1. Data Quality Control

Data quality is a major barrier to the clinical use of AI in PH. Most current models are developed from retrospective or single-center datasets, with differences in patient selection, imaging protocols, diagnostic labels, and outcome definitions. This is particularly important in PH because right heart catheterization remains the diagnostic gold standard, whereas many AI studies use surrogate labels such as echocardiographic estimates, imaging signs, diagnostic codes, or claims-based definitions. Tertiary referral cohorts and datasets enriched for suspected or established PH may also introduce spectrum and selection bias, potentially inflating apparent performance relative to lower-prevalence community or primary-care populations. Existing PAH registries provide valuable clinical data, but they often lack complete multimodal information, including imaging, omics, longitudinal treatment response, and patient-reported outcomes [105]. Some PAH diagnostic models also highlight risks of bias, limited external validation, and uncertain generalizability [106,107]. In addition, AI-based extraction of right heart catheterization reports may improve data collection, but extraction errors and missing values still require manual quality control [108].

PH-AI studies are also vulnerable to model-development biases. Small datasets combined with high-dimensional imaging or omics features increase the risk of overfitting, while inappropriate splitting of repeated examinations from the same patient across training and test sets can cause data leakage and overly optimistic performance estimates. Class imbalance is another concern because specific PH groups and clinically important adverse outcomes may be substantially less frequent than comparator classes. Patient-level data partitioning, prespecified evaluation sets, appropriate handling of class imbalance, transparent model selection, and independent external evaluation should therefore be considered essential components of model assessment. Therefore, future PH-AI research should rely on multicenter datasets, standardized RHC-confirmed labels, harmonized imaging protocols, and transparent reporting frameworks such as PRIME 2.0 [109].

#### 6.2.2. Poor Technology Design

Poor technology design limits clinician trust and real-world adoption. Many PH-AI models report high diagnostic or prognostic performance, but their outputs are often difficult to interpret. This is particularly important because PH management involves high-stakes decisions, including referral for RHC, targeted therapy, BPA, PEA, and transplant evaluation. Explainability methods such as SHAP, Grad-CAM, feature attribution, and attention maps can indicate which variables or image regions contribute to model predictions and may help identify unexpected model behavior [42,110,111]. However, such explanations should not be interpreted as evidence that a model is clinically reliable or that the highlighted features represent causal or clinically meaningful relationships [111]. Reliability requires complementary evaluation of calibration, uncertainty, failure modes, and performance across clinically relevant subgroups, centers, devices, and acquisition settings. Future PH-AI systems should therefore combine explainability with uncertainty estimation, calibration assessment, subgroup analysis, and external validation rather than relying on visual or feature-based explanations alone [109,110,111].

#### 6.2.3. Lack of Clinical Integration

The current evidence for AI in PH spans different stages of clinical maturity that should not be considered equivalent. Many models have been evaluated retrospectively using internal validation, demonstrating technical feasibility but providing limited evidence of generalizability, whereas a smaller subset has undergone independent external validation [2,106,107]. External validation provides stronger evidence that model performance may generalize across populations or institutions but does not by itself establish clinical benefit. Evidence from prospective studies in which AI systems are embedded into actual clinical workflows remains limited [2,107,109].

For example, CXR-based and ECG-based models may identify patients at increased risk of PH [4,6,11,12,13,14,85,90], but their clinical value ultimately depends on whether their use improves subsequent diagnostic evaluation, PH-center referral, or selection for RHC. Similarly, CT-based, CMR-based, and echocardiography-based AI should ultimately be integrated and evaluated within PACS, EHR systems, reporting workflows, and multidisciplinary decision-making rather than assessed only as stand-alone algorithms [2,107,109]. Before routine implementation, AI-supported pathways require prospective evaluation of calibration, safety, subgroup performance, clinician interaction, workflow efficiency, and downstream clinical consequences [109]. For screening systems, this includes establishing clinically appropriate referral thresholds and acceptable false-positive and false-negative rates; for treatment-support systems, evidence is needed that AI-guided recommendations improve clinical decisions or patient outcomes beyond established approaches. Prospective workflow and impact studies, together with appropriate post-deployment monitoring, are therefore required before routine clinical use [2,109].

Clinical implementation also requires assessment of net benefit and health-economic consequences. Even a well-discriminating screening model may have limited value if false-positive classifications substantially increase specialist referrals, repeat echocardiography, advanced imaging, or unnecessary RHC. Prospective implementation studies should therefore evaluate diagnostic yield, false-positive referral burden, downstream testing, resource utilization, cost-effectiveness, and patient-important outcomes in addition to conventional accuracy metrics.

#### 6.2.4. Privacy Concerns

AI applications in PH often require sensitive multimodal data, including imaging, ECG, echocardiography, right heart catheterization records, clinical notes, genomic data, drug exposure, and long-term outcomes. Because PH is rare, multicenter collaboration is necessary, but data sharing increases privacy risks. Privacy-preserving methods such as federated learning may allow institutions to train models collaboratively without directly exchanging raw patient data. A CT pulmonary vessel segmentation study using local and federated learning illustrates this direction [112]. However, PH-specific evidence remains limited and federated learning should currently be regarded as an emerging methodological strategy rather than an established clinical solution. Generative AI and automated text extraction also raise privacy concerns, since clinical reports contain identifiable and sensitive information [108]. Future PH-AI systems should therefore include de-identification, secure storage, access control, audit trails, clear consent procedures, and privacy-preserving model development.

#### 6.2.5. Non-Adherence

AI tools may fail if clinicians or patients do not use them consistently. Clinicians may distrust black-box outputs, especially when predictions conflict with clinical judgment. Patients may also feel burdened by mobile apps, wearable devices, remote monitoring, or repeated symptom reporting. AI and mobile health may support PAH monitoring and long-term management, but their success depends on usability, digital literacy, patient engagement, and clinician supervision [113]. Telehealth systems can support chronic disease follow-up, but they require sustained adherence and access to digital devices [114]. Patient-facing AI must be used carefully, as studies of ChatGPT responses to PAH-related questions found potential value but also concerns about readability and safety [115]. Thus, AI tools should be simple, transparent, clinician-supervised, and designed around real patient needs.

#### 6.2.6. Imperfection of Laws and Regulations

Regulatory and legal frameworks for AI applications in PH are still incomplete. Key unresolved issues include validation standards, responsibility for AI-assisted errors, post-deployment monitoring, data privacy, reimbursement, and model updating. These issues are especially important in PH because AI outputs may influence invasive testing, targeted therapy, interventional procedures, transplant referral, and monitoring intensity. PRIME 2.0 provides a useful framework for evaluating AI models of multimodal cardiovascular imaging and may guide future PH studies [109]. Transparency is also essential for hospital acceptance and clinical accountability [116]. In addition, AI-based pharmacovigilance may help detect drugs associated with PAH, but differences among real-world data sources show that safety signals require careful verification [117,118]. Future regulations should define standards for external validation, explainability, bias assessment, clinical responsibility, and post-market surveillance before AI tools are widely used in PH care. (The challenges and solutions mentioned above are summarized in Table 4).

## 7. Conclusions and Perspective

### 7.1. Clinically Promising Applications of AI in Pulmonary Hypertension

Current evidence suggests that the most clinically promising AI applications in PH are those built on routinely available data and clearly linked to diagnostic or management decisions. Chest radiography-based AI is a representative example. Because chest X-ray is inexpensive, widely available, and frequently performed in primary care, emergency departments, and preoperative assessment, AI-assisted interpretation may help detect patients who otherwise would not be referred for further PH evaluation. Deep learning models have been applied to suspected PH, PAH, systemic sclerosis-associated PH, congenital heart disease-associated PAH, and exercise-induced PH, with several studies reporting good diagnostic performance and potential value in low-resource settings [4,5,6,8,90].

ECG-based AI provides another scalable approach. Standard 12-lead ECG is easy to obtain, but conventional ECG interpretation often lacks sensitivity for early PH. Deep learning models using ECG voltage-time signals can detect PH, elevated pulmonary pressure, and clinically relevant PH subtypes, and some algorithms retain predictive ability months or even years before formal diagnosis [11,12,13,85]. These findings support the use of ECG-AI as an upstream screening tool rather than a replacement for echocardiography or right heart catheterization.

Echocardiography-based AI is closer to routine PH assessment because ultrasound is already the first-line tool for estimating PH probability and evaluating right heart structure. AI models have been used for automated tricuspid regurgitation jet velocity measurement, PH probability estimation, differentiation of pre-capillary and post-capillary PH, right ventricular functional assessment, and interpretable chamber-based PAH diagnosis [39,41,42,46,75]. These tools may reduce operator dependence and improve consistency, especially in centers without dedicated PH expertise.

CT-based and CMR-based AI further extend PH evaluation from detection to quantitative phenotyping and risk assessment. CT-based models can segment pulmonary vessels and lung parenchyma, evaluate chronic thromboembolic lesions, quantify perfusion abnormalities, and support PH subtype classification [17,18,20,72,119]. CMR-based AI can automatically quantify right ventricular and right atrial structure and function, link these measures with invasive hemodynamics, and predict clinical outcomes in PAH and suspected PH [37,38,92]. More recent approaches have extended CMR-based analysis toward noninvasive hemodynamic estimation, including machine-learning prediction of pulmonary hemodynamics and physics-informed modeling of pulmonary artery pressure from magnetic resonance imaging [119,120,121]. These examples show that AI is most useful when it converts complex imaging information into reproducible, clinically interpretable variables.

Multimodal AI may better reflect real-world PH decision-making than single-modality algorithms. Models integrating ECG, chest radiography, BNP, echocardiography, clinical variables, and EHR data have improved PH detection and may help clinicians decide which patients require right heart catheterization, or specialist referral [9,14,44,86]. Moreover, experience from other areas of cardiology also provides useful translational context. In cardiac electrophysiology, AI has been applied to ECG interpretation, arrhythmia detection, atrial mapping, and catheter-ablation workflows, illustrating how AI can extend from automated signal analysis toward procedure-oriented clinical decision support [122]. These examples support the broader feasibility of clinically integrated cardiovascular AI, although differences in disease biology, prevalence, diagnostic pathways, and available data mean that performance and implementation experience should not be directly extrapolated to PH. Taken together, successful AI applications in PH are likely to be accessible, interpretable, externally validated, and embedded into clinical workflows rather than used as isolated diagnostic tools.

### 7.2. Recommendations for Future Directions

Future research about AI in PH should first focus on data quality and external validation. Many current studies remain retrospective, single-center, and limited by small sample size, inconsistent labels, missing data, and incomplete representation of rare PH subtypes. Since PH diagnosis relies on right heart catheterization, future datasets should prioritize standardized RHC-confirmed labels while also incorporating ECG, CXR, CT, CMR, echocardiography, laboratory tests, medications, longitudinal outcomes, and treatment-response data. PAH registries and diagnostic accuracy reviews have emphasized the need for better data structure, bias control, and generalizability assessment. Future prediction-model studies should also follow contemporary reporting and appraisal frameworks, including TRIPOD + AI for transparent reporting of prediction-model development and evaluation [123] and PROBAST + AI for assessment of model quality, risk of bias, and applicability [124], together with domain-specific frameworks such as PRIME 2.0 for cardiovascular imaging and multimodal AI [109].

Second, future studies should move beyond internal performance metrics. High AUC values are not sufficient if a model fails across hospitals, imaging vendors, disease subgroups, or clinical workflows. Prospective validation should test whether AI-assisted pathways actually reduce diagnostic delay, improve referral accuracy, reduce unnecessary invasive testing, guide treatment escalation, or improve patient outcomes. This is especially important for AI models used in high-stakes PH decisions, including right heart catheterization referral, PAH-targeted therapy, BPA, PEA, transplant evaluation, and right heart failure monitoring.

Third, explainability should be considered alongside, rather than as a substitute for, model reliability. Methods such as Grad-CAM, SHAP, attention maps, and feature attribution can help clinicians understand which image regions or variables influence a prediction, but they do not establish that these associations are causal, clinically meaningful, or generalizable. Explainable outputs should therefore be accompanied by calibration assessment, uncertainty estimation, failure detection, subgroup performance analysis, and external validation. This distinction is particularly important in high-stakes PH decisions, where a plausible explanation cannot compensate for an unreliable or poorly calibrated model.

Fourth, AI systems should shift from single-task prediction toward integrated decision support. Instead of only asking whether PH is present, future models should help answer clinically actionable questions: who should be screened, which PH subtype is most likely, whether the patient is deteriorating, which treatment option is most appropriate, and how follow-up intensity should be adjusted. This requires integration of diagnosis, classification, prognosis, treatment-response prediction, and right heart failure assessment. Doctor-AI cooperative platforms, computational simulators, response-prediction models, and RV phenotyping tools provide early examples of this direction.

Fifth, privacy-preserving and collaborative learning should be encouraged. PH is uncommon and heterogeneous, so single-center datasets are unlikely to support robust models for all subgroups, especially pediatric PH, congenital heart disease-associated PAH, CTEPH, systemic sclerosis-associated PAH, and omics-defined phenotypes. Federated learning, distributed analytics, secure de-identification, and clear data governance may allow institutions to collaborate while protecting patient privacy [112]. These strategies are particularly relevant for imaging, genomic, EHR, and long-term monitoring data.

Finally, clinical implementation should be patient-centered, clinician-supervised, and responsive to evolving regulatory requirements. AI tools should undergo usability testing, bias assessment, and post-deployment monitoring, with clear delineation of clinical responsibility and mechanisms for clinician oversight. These safeguards are particularly important for patient-facing applications, including mobile health systems and large language models, where inaccurate information, poor readability, or inappropriate reassurance may adversely affect adherence, healthcare-seeking behavior, and patient safety. Accordingly, AI should be integrated into PH care as decision support that complements established clinical pathways rather than as a source of autonomous medical advice. This cautious approach is especially important when considering emerging AI technologies, for which the maturity of evidence varies substantially. Large language models may facilitate information extraction, summarization, and patient communication, but PH-specific evidence regarding prospective safety, clinical effectiveness, and workflow integration remains limited. Federated learning offers a potential strategy for multicenter model development while reducing the need for direct exchange of raw patient data, although its application in PH remains at an early stage. Digital twins and patient-specific computational simulation are even less mature and should currently be regarded primarily as research frameworks rather than validated tools for treatment selection. Thus, while these technologies may address important needs in future PH care, their transition from proof-of-concept applications to routine clinical use will require rigorous prospective evaluation, clearly defined clinical roles, and appropriate regulatory and governance frameworks.

### 7.3. Construction of an AI-Assisted Digital Health-Care Ecosystem for Pulmonary Hypertension Management

The future goal of AI in PH should not be the creation of separate diagnostic algorithms, but the development of an integrated digital healthcare ecosystem covering the entire patient journey. Importantly, such an ecosystem remains a prospective framework rather than an established model of PH care. Current studies have demonstrated individual components, including AI-assisted analysis of ECG, chest radiography, echocardiography, CT, and CMR; automated imaging quantification; risk prediction; and selected multimodal models. However, continuous deterioration prediction, AI-guided treatment optimization, longitudinal multimodal integration, and closed-loop model updating have not yet been established as routine clinical capabilities in PH.

Within this future framework, low-burden community and primary-care screening could use routinely available ECG, chest radiography, digital stethoscope recordings, BNP testing, and EHR-based risk models to identify patients who may require further evaluation in dyspnea clinics or specialized PH centers. At the diagnostic stage, AI-assisted echocardiography, CT, CMR, and hemodynamic analysis could integrate complementary information on pulmonary pressure, right ventricular function, pulmonary vascular morphology, lung parenchymal disease, thromboembolic lesions, and PH subtype, while final diagnosis and treatment decisions would remain under expert clinical supervision and RHC when indicated.

At the risk-stratification and treatment stage, future AI systems could integrate functional capacity, biomarkers, imaging, hemodynamics, comorbidities, omics data, and previous treatment response to generate individualized risk profiles and potentially support treatment selection or escalation. Although prognostic and treatment-response models provide early proof of concept, evidence that AI-guided treatment recommendations improve therapeutic decisions or patient outcomes remains insufficient. Similarly, longitudinal integration of repeat imaging, laboratory results, EHR data, wearable signals, patient-reported symptoms, and medication adherence could potentially support earlier detection of deterioration, but continuous AI-based monitoring has not yet been prospectively validated as a standard PH management strategy.

Closed-loop learning represents a further prospective stage in which real-world outcomes could be used to update risk prediction, refine phenotypes, and identify model drift. Its implementation would require robust data interoperability, prospective multicenter validation, predefined model-update procedures, continuous calibration and drift monitoring, privacy and cybersecurity safeguards, regulatory oversight, clinician accountability, and post-deployment surveillance. Thus, the proposed ecosystem should be regarded as a roadmap for future clinical translation rather than a description of currently established PH care. AI should remain clinician-supervised, with implementation guided by evidence that it improves workflow, clinical decisions, and ultimately patient outcomes.

## Figures and Tables

**Table 1:** Methodological characteristics, validation strategies, performance, and limitations of representative AI studies for PH detection and assessment.

Study	Modality/Clinical Task	Sample Size	Reference Standard/Disease Label	Validation Approach	Main Performance	Major Limitations
Han et al. [3]	CXR; CHD and CHD-associated PAH detection	3255 CXRs	Clinical diagnosis of CHD/PAH-CHD	Fivefold cross-validation; multi-reader multi-case test	AUC 0.948 for CHD; 0.778 for CHD-associated PAH	Retrospective; disease-specific preoperative population; CHD and CHD-associated PAH represent different classification tasks
Zou et al. [4]	CXR; PH screening	762 participants	PASP estimated by Doppler echocardiography	8-fold cross-validation; internal and external test sets	AUC 0.970 internal; 0.967 external	Retrospective; echocardiographic rather than invasive reference standard; small external test set
Kusunose et al. [5]	CXR; exercise-induced PH in SSc/CTD	142 patients	Exercise stress echocardiography; exercise RHC in a subset	Internal model evaluation	AUC 0.82	Small, selected SSc/CTD cohort; exercise-induced PH differs from resting PH; limited invasive confirmation
Imai et al. [6]	CXR; PAH detection	519 CXRs from 405 patients	Established PAH diagnosis	Training and held-out test sets	AUC 0.988; sensitivity 0.933; specificity 0.982	Single-center retrospective study; repeated images from some patients; no independent external validation
Shimbo et al. [7]	CXR; PH detection in SSc	230 patients	RHC	Internal model evaluation	AUC 0.826	Retrospective; small disease-specific cohort; no independent external validation
Li et al. [8]	CXR; PAH risk in children with VSD	831 patients	Occurrence of PAH in VSD	Validation and independent test sets	AUC 0.82 validation; 0.81 independent test	Retrospective single-center pediatric VSD cohort; endpoint differs from general PH detection
Kishikawa et al. [9]	ECG + CXR + BNP; PH detection	71,826 patients; 123,260 ECGs	Study-defined PH diagnosis	Patient-level training/validation/test split	AUC 0.818 ECG; 0.823 CXR; 0.724 BNP; 0.872 combined	Retrospective; no independent external cohort; multimodal performance not directly comparable with single-modality models
DuBrock et al. [11]	ECG; PH detection/early recognition	259,227 Mayo patients; external cohort 30,301	EHR-derived PH-likely definition incorporating clinical diagnostic data	Mayo development/testing; independent external validation at Vanderbilt	AUC 0.920 Mayo; 0.880 external	Retrospective EHR-based phenotyping; diagnostic labels not uniformly based on contemporaneous RHC
Liu et al. [12]	ECG; PH detection and mortality prediction	41,097 patients; external cohort 279	ECG paired with echocardiographic assessment	Patient-level 10-fold cross-validation; independent external validation	AUC 0.880 internal and 0.880 external	Retrospective; predominantly echocardiography-based labeling; small external cohort
Aras et al. [13]	ECG; PH and subtype detection	24,470 patients	RHC or echocardiography within 90 days	Patient-level 7:1:2 train/development/test split	AUC 0.890 for overall PH	Retrospective single-center study; mixed RHC/echocardiographic reference standards; no external validation
Liu et al. [14]	ECG + CXR; PAH detection	Study-specific development and external cohorts	Study-defined PAH criteria	Internal and independent external validation	AUC 0.8644 internal; 0.8734 external; NPV >98%	Retrospective; multimodal inputs; cohort composition and task differ from ECG- or CXR-only models
Sharkey et al. [16]	CTPA; cardiac chamber/great-vessel segmentation	Multicohort CTPA dataset	Expert manual segmentation	Internal and external test cohorts	High DSC for most cardiovascular structures	Segmentation rather than direct PH diagnosis; performance depends on image quality and acquisition characteristics
Xu et al. [17]	CTPA; differentiation of CTEPH, CTED, and controls	190 participants: 116 CTEPH, 54 CTED, 20 controls	Clinical/hemodynamic classification	Retrospective cohort analysis/ROC evaluation	PA tortuosity AUC 0.859 for CTEPH vs. controls; 0.663 for CTEPH vs. CTED	Retrospective; small control group; modest discrimination between CTEPH and CTED; no external validation
Silva et al. [19]	CTPA; pulmonary embolism detection	800 RSNA exams for model development; 216 external exams	CTPA/report-based PE labels	Internal validation plus independent real-world external validation	Internal exam-level accuracy 77%; external precision 86% positive/69% negative	PE rather than established PH; evidence is indirectly relevant to PH/CTEPH workflows; signs of overfitting in internal validation
Cereser et al. [20]	Contrast-enhanced CT; PH group classification	172 PH patients	Expert-reader consensus based on CT features and ESC/ERS groups	Retrospective two-center dataset; algorithm comparison	Accuracy 0.72; AUROC 0.84; F1 0.72	Small cohort; expert-reader-derived rather than hemodynamic ground truth; limited Group V representation; no independent external validation
Swift et al. [35]	CMR; PAH diagnosis	220 patients: 150 PAH, 70 no PH	RHC and multidisciplinary diagnosis	Internal model evaluation	AUC 0.92	Retrospective referral-center cohort; selected PAH-vs-no-PH task; no independent external validation
Lungu et al. [36]	CMR + computational hemodynamics; PH diagnosis	72 patients: 57 PH, 15 no PH	RHC	Leave-one-out cross-validation	Accuracy 92%; AUC ≈ 0.91 for combined model	Small, imbalanced single-center cohort; cross-validation only; no external validation
Alabed et al. [37]	CMR; automated ventricular measurements and prognostic assessment	Large multicohort PH dataset	Manual CMR measurements; invasive hemodynamics and mortality	Independent clinical testing and scan-rescan assessment	High agreement/repeatability; associations with invasive hemodynamics and mortality	Primarily automated quantification rather than direct PH diagnosis; specialized CMR cohorts
Alandejani et al. [38]	CMR; automated RA measurements	365 training; 36 prospective repeatability; 400 clinical testing; 3795 QC	Manual CMR; RHC in 212/400; mortality	Multicohort training, prospective repeatability and independent clinical testing	ICC 0.91–0.95; AUC 0.82/0.87 for RAP thresholds	Primarily measurement/risk assessment rather than PH detection; referral-center population
Leha et al. [39]	Echocardiography; PH prediction	Study-specific clinical cohort	Clinical/echocardiographic PH assessment	Cross-validation/internal evaluation	AUC 0.87 for best random-forest model	Single-study cohort; conventional echo variables rather than image-based end-to-end AI; limited external validation
Liao et al. [40]	Echocardiography; PH probability	346 patients including 275 in development and 71 in external validation	RHC within 24 h	Internal plus external validation at two independent hospitals	AUC 0.945 development; 0.950 external	Retrospective; selected suspected-PH population; model based primarily on parasternal short-axis images
Salehi et al. [41]	Echocardiography; automated TRJV/PH detection	1031 suspected-PH patients	RHC; mPAP > 20 mmHg	Retrospective clinical validation	AUC 0.88; TRJV measurable in 87%	Focused on automated TRJV rather than comprehensive PH diagnosis; performance dependent on measurable TR signal
Sun et al. [42]	Echocardiography; interpretable PAH detection	13,912 development subjects; 3150 external test subjects	Echocardiographic/report-based PAH labels	Internal plus independent external testing	External AUC 0.862 for A4C-based model	Non-RHC reference standard; retrospective; class imbalance; view-dependent performance
Luo et al. [43]	Echo + CXR; PAH after VSD correction	Pediatric VSD cohort	Clinical diagnosis of postoperative PAH	Internal and external testing	AUC, accuracy, and F1-score > 0.90	Highly selected pediatric postoperative VSD population; multimodal task not directly comparable with general PH detection

Abbreviations: A4C, apical four-chamber view; AI, artificial intelligence; AUC/AUROC, area under the receiver operating characteristic curve; BNP, brain natriuretic peptide; CHD, congenital heart disease; CMR, cardiac magnetic resonance; CT, computed tomography; CTED, chronic thromboembolic disease; CTEPH, chronic thromboembolic pulmonary hypertension; CTPA, computed tomography pulmonary angiography; CTD, connective tissue disease; CXR, chest radiography; DSC, Dice similarity coefficient; ECG, electrocardiography; ESC/ERS, European Society of Cardiology/European Respiratory Society; ICC, intraclass correlation coefficient; mPAP, mean pulmonary artery pressure; NPV, negative predictive value; PA, pulmonary artery; PAH, pulmonary arterial hypertension; PASP, pulmonary artery systolic pressure; PE, pulmonary embolism; PH, pulmonary hypertension; QC, quality control; RA, right atrium; RAP, right atrial pressure; RHC, right heart catheterization; ROC, receiver operating characteristic; RSNA, Radiological Society of North America; SSc, systemic sclerosis; TR, tricuspid regurgitation; TRJV, tricuspid regurgitation jet velocity; VSD, ventricular septal defect.

**Table 2:** Precision phenotyping approaches and their potential clinical relevance in PH.

Phenotyping Layer	Data Examples	AI Approach	Potential Clinical Relevance	Evidence Status	References
Guideline-based phenotype	Clinical group, RHC pattern, CT thromboembolic signs, left-heart disease indicators	Supervised classifiers; expert-feature ML	May support initial classification and referral while preserving RHC and expert confirmation	Guideline anchored, but AI support remains adjunctive and requires clinical/RHC confirmation	[1,20,46,47]
Comorbidity phenotype	Kidney disease, heart disease, interstitial lung disease, COPD/emphysema	Clustering; clinical phenomapping; EHR feature extraction	Identifies patterns of systemic comorbidity associated with risk and may support referral prioritization	Exploratory to replicated observational evidence; prospective impact evaluation remains limited	[53,73,74]
RV-centered phenotype	RV-PA coupling, contractility, stiffness, RV/RA size and function, CMR/echo markers	Deep phenotyping; hemodynamic clustering; automated segmentation	Characterizes adaptive and maladaptive right-heart remodeling and prognostically relevant RV heterogeneity	Observational and prognostic; clinical utility depends on linkage to RV-PA load and patient outcomes	[37,38,62,64,65,75,76]
Imaging phenotype	CT perfusion, vessel tortuosity, parenchymal patterns, pulmonary blood volume, fibrosis	Radiomics; segmentation; automated quantitative imaging; Bayesian analysis	Supports quantitative characterization of vascular, parenchymal, and perfusion abnormalities and may assist disease phenotyping	Predominantly retrospective quantitative-imaging evidence; external validation varies by task	[16,17,18,66,67,68,69,70,71,72,73,77]
Molecular /immune endotype	Whole-blood transcriptomics, cytokines, metabolomics, proteomics, microbiome metabolites	Unsupervised clustering; pathway ML; biomarker-panel selection	Identifies biologically distinct subgroups associated with disease mechanisms and prognosis; therapeutic relevance remains investigational	Primarily exploratory or associative; independent biological and clinical replication remains limited	[50,55,56,57,58,59,78,79,80]
Therapy-response phenotype	Baseline risk, hemodynamics, echo/CT features, prior therapies, real-world outcomes	Survival forests; response prediction; computational simulation; SHAP interpretation	May inform treatment-response stratification, but clinical utility for treatment selection remains to be prospectively established	Proof of concept; prospective treatment-selection utility has not been established	[45,51,57,81,82,83,84]

Abbreviations: AI, artificial intelligence; CMR, cardiac magnetic resonance; COPD, chronic obstructive pulmonary disease; CT, computed tomography; EHR, electronic health record; ML, machine learning; PA, pulmonary artery; PH, pulmonary hypertension; RA, right atrium; RHC, right heart catheterization; RV, right ventricle; SHAP, SHapley Additive exPlanations.

**Table 3:** AI applications across the PH clinical pathway.

Care Stage	Unmet Clinical Need	AI-Supported Data Sources	Potential Output	Evidence Requirement
Screening/prevention	Delayed recognition due to nonspecific symptoms and limited PH-center access	ECG, CXR, EHR, claims, BNP, digital stethoscope	High-risk flag; referral suggestion; proactive surveillance list	Compare with existing screening/referral pathways and quantify prevalence-dependent PPV/NPV and false-positive referrals
Diagnosis	RHC is definitive but invasive; noninvasive tests may be operator-dependent	Echo, CXR, ECG, CT/CTPA, CMR, RHC reports	PH probability; elevated PAP prediction; right-heart burden estimate	Compare with the guideline diagnostic pathway, preserve RHC confirmation when indicated, and quantify downstream testing
Guideline classification	Overlap among PH groups; difficulty distinguishing pre- and post-capillary PH	Echo variables, CT features, clinical data, hemodynamics	Likely PH clinical group; referral triage; specialist review support	Compare with expert guideline classification and demonstrate clinically useful reclassification
Refined phenotyping	Patients within the same group differ in prognosis and therapy response	Imaging, hemodynamics, comorbidities, biomarkers, multi-omics	RV phenotype; immune/metabolic endotype; high-risk phenogroup	Demonstrate reproducibility across independent cohorts and biological/clinical validity
Risk and prognosis	Need individualized mortality, hospitalization, and clinical-worsening estimates	CMR, CT, ECG, echo, biomarkers, functional capacity, EHR	Survival risk; readmission risk; RV failure warning; risk driver explanation	Compare directly with ESC/ERS and REVEAL-based tools and assess calibration, reclassification, and clinical impact
Treatment planning	Trial-and-error therapy and uncertain response to BPA/PEA/drugs	Clinical/echo, CT/CTPA perfusion, biomarkers/omics, prior response, claims	Treatment-response prediction; escalation suggestion; clinical trial matching	Demonstrate treatment-by-model interaction and improved decisions or outcomes prospectively
Monitoring	Need reproducible longitudinal measurement and early deterioration detection	Repeat CMR/echo/CT, labs, RHC text, wearables, patient-reported symptoms	Dynamic risk update; remote alert; reproducible measurement; deterioration warning	Demonstrate earlier actionable deterioration detection, acceptable alert burden, and improved outcomes

Abbreviations: AI, artificial intelligence; BNP, brain natriuretic peptide; BPA, balloon pulmonary angioplasty; CMR, cardiac magnetic resonance; CT, computed tomography; CTPA, computed tomography pulmonary angiography; CXR, chest radiography; ECG, electrocardiography; EHR, electronic health record; ESC/ERS, European Society of Cardiology/European Respiratory Society; NPV, negative predictive value; PAP, pulmonary artery pressure; PEA, pulmonary endarterectomy; PH, pulmonary hypertension; PPV, positive predictive value; REVEAL, Registry to Evaluate Early and Long-term Pulmonary Arterial Hypertension Disease Management; RHC, right heart catheterization; RV, right ventricle.

**Table 4:** Challenges and proposed solutions for clinical implementation of AI in PH.

Implementation Challenge	Importance in PH	Practical Solution	References
Data quality control	PH is rare and heterogeneous; labels may rely on echo, codes, or claims rather than RHC	Use multicenter cohorts, RHC-confirmed labels, harmonized imaging protocols, missing-data plans Include patient-level splitting, leakage prevention, class-balance reporting, and external evaluation in intended-use populations	[105,106,107,109]
Interpretability and robustness	Difficult-to-interpret outputs can undermine trust in high-stakes decisions such as RHC referral, targeted therapy, BPA/PEA, and transplant evaluation	Use SHAP/Grad-CAM/attention maps, uncertainty estimation, failure detection Report calibration, uncertainty, subgroup performance, failure analysis, and clinician override	[42,110,111]
Workflow integration	High model performance has limited value unless results trigger timely echo, RHC, PH-center referral, or other clear next steps	Integrate AI into EHR/PACS and reporting workflows; provide actionable alerts and clinician override Evaluate diagnostic yield, referral appropriateness, downstream testing, workflow time, and clinical outcomes	[2,107,109]
Privacy and governance	Sensitive data include imaging, ECG, RHC, notes, genomics, drug exposure, and outcomes	Use federated learning, de-identification, secure storage, access control, audit trails, and clear consent Audit privacy, cross-site performance, governance compliance, and secure update procedures	[108,112]
Adherence and usability	Clinicians may distrust black-box tools; patients may be burdened by apps, wearables, or repeated reporting	Use clinician supervision, readable patient-facing outputs, usability testing, and digital-literacy adaptation Assess adoption, alert burden, readability, digital equity, and sustained clinician/patient use	[113,114,115]
Regulation and accountability	Standards remain incomplete for validation, responsibility, privacy, reimbursement, model updating, and post-deployment monitoring	Define external validation, explainability, bias, responsibility, update, reimbursement, and post-market surveillance requirements Include cost-effectiveness, reimbursement, accountability, drift monitoring, and post-market surveillance	[109,116,117,118]

Abbreviations: AI, artificial intelligence; BPA, balloon pulmonary angioplasty; ECG, electrocardiography; EHR, electronic health record; Grad-CAM, gradient-weighted class activation mapping; PACS, picture archiving and communication system; PEA, pulmonary endarterectomy; PH, pulmonary hypertension; RHC, right heart catheterization; SHAP, SHapley Additive exPlanations.

## Data Availability

No new data were created or analyzed in this study. Data sharing is not applicable to this article.
